# Book Review: Cognitive Behavioral Therapy Made Simple: 10 Strategies for Managing Anxiety, Depression, Anger, Panic, and Worry

**DOI:** 10.3389/fpsyg.2021.749259

**Published:** 2021-10-26

**Authors:** Mohamad Farhan Huszaimi Bin M. Pajar

**Affiliations:** ^1^Department of Neuroscience, Universiti Sains Malaysia, Kelantan, Malaysia; ^2^Ampang Hospital, Ministry of Health, Ampang, Malaysia

**Keywords:** cognitive behavior therapy (CBT), anxiety, depression, psychotherapy, stress, behavioral activation (BA), mindfulness, anger management

Cognitive Behavioral Therapy (CBT) is well-known among clinical practitioners as one of the most commonly used types of psychotherapy (Hofmann et al., 2012). However, a specific CBT technique was discovered to be more effective in managing some symptoms of one specific psychopathology. Dr. Seth J. Gillihan, an assistant professor in the Psychiatry Department at the University of Pennsylvania and a licensed registered psychologist in the United States, published this book intended for general readers to help them cope with anxiety-related symptoms, depression, anger, and concern through utilizing certain CBT psychotherapy techniques supported by scientific evidence, such as behavior activations, challenging thinking processes, and mindfulness.

The book is divided into 11 chapters. Chapter one discusses a very brief history of psychotherapy and the development history of CBT by combining behavioral therapy and cognitive therapy, which helps the audience understand how CBT was formed and why it is helpful. The book is also up to date on current psychotherapy trends, with the author incorporating the concept of mindfulness into CBT practice. The author goes on to describe the eight principles of CBT, as well as giving a general explanation of how CBT can help in symptom reduction, particularly for depression, anxiety, and stress, which is important in assisting the audience's understanding of how CBT can help them.

In addition, Chapter two discusses goal setting. The author mentions the benefits of having goals, as well as goal-setting techniques such as (1) Be Specific, (2) Finding the Right Pace, and (3) The Value of Achieving the Goal. Goal setting can be hard for most people, especially those who suffer from depressive symptoms, anxiety-related symptoms, or stress (Watkins, 2011); as a result, the author offers some suggestions, such as relationships, meaning, education or work, physical health, and recreation. The suggestions may be useful for general readers or clinicians seeking ideas for activating their own or their clients' behaviors. The author then describes the brain anatomies responsible for mood (Fuchs and Flugge, 2003), which may help readers understand the brain's role in stress. Chapter Three is about behavioral activation, which is also known as one of the fundamental techniques used in CBT. The chapter explains the benefit of behavioral activation for mood improvement, strategies for determining types of behavior, certain techniques for setting behavioral goals, and techniques to remain motivated to help practice the intended behavior which can provide ideas for both public readers and for clinicians to use with clients during sessions.

Furthermore, Chapter Four focuses on recognizing and breaking negative thought patterns. The early part explains how thoughts affect one's emotions and gives methods for identifying problematic thoughts, by providing a list of common thinking errors (p. 60) with examples to help readers identify their own cognitive distortions. The following Chapter Five is on how to identify and challenge one's core belief, which is how a person views themselves. The book provides two simple techniques for identifying core beliefs that are easily understood by the audience, as well as three methods for changing core beliefs. Mindfulness, also known as the “third wave” of current psychotherapy approaches, is discussed in Chapter Six. The author begins by introducing the concept of mindfulness, which entails being present in the moment and accepting the experience (p. 88–89). Different books define mindfulness in different ways, but because the author's target audience is broad, Dr. Gillihan attempts to define mindfulness in the most basic terms possible. In layman's terms, the author also debunks some mindfulness myths and explains how to actually be mindful.

Chapters seven through nine focus on specific issues such as procrastination (Chapter Seven), worry, fear, and anxiety management (Chapter Eight), and anger management (Chapter Nine). The author discusses strategies for overcoming procrastination by utilizing the three basic pillars of CBT covered in previous chapters: (1) Think—which is to analyze thought, (2) Act—to modify behaviors, and (3) Be—to represent being mindful of the present moment (p. 105). The author begins by explaining the differences between worry, fear, and anxiety in layman's terms so that the reader could differentiate between those concepts. Each type of anxiety, such as generalized anxiety disorder (GAD) and panic disorder, is summarized. However, the author has included post-traumatic stress disorder (PTSD) and obsessive-compulsive disorder (OCD) together with the topic, despite the fact that PTSD and OCD should be classified separately because the *Diagnostic and Statistical Manual of Mental Disorders fifth* edition *(DSM-5)* classifies PTSD and OCD separately from anxiety disorders (American Psychiatric Association, 2013). The author then discusses how to manage anger using the same technique, which is to think, act, and be mindful. The book comes to a close with Gillihan reminding the reader to be kind to themselves, which is also a mindfulness concept, and a conclusion about the overall use of the book. The author also reminds readers to seek professional help if they feel it necessary, with a reference based on a certified therapist in the United States.

Finally, this book is intended for general readers who are unfamiliar with CBT or psychotherapy. many people might find the book extremely beneficial because it covers the fundamental skills needed to cope with stressors such as depression, anxiety, and stress. The book will also be beneficial to those who are currently working with a professional mental health therapist to practice CBT. The book can also be used as a reference, source of ideas, or guide for clinical professionals to explain concepts to patients in layman's terms. Readers with diverse backgrounds will benefit from this book because each chapter concludes with a summary and activities in which readers can participate to directly apply that chapter's concept, allowing them to master the skill of CBT. Hence, I believe this book is appropriate for anyone: professional clinicians, non-professionals in mental health, and general public alike, with or without any mental health issues.

## Author Contributions

MP was the only author for this publication and work has been done independently throughout the manuscript production process.

## Conflict of Interest

The author declares that the research was conducted in the absence of any commercial or financial relationships that could be construed as a potential conflict of interest.

## Publisher's Note

All claims expressed in this article are solely those of the authors and do not necessarily represent those of their affiliated organizations, or those of the publisher, the editors and the reviewers. Any product that may be evaluated in this article, or claim that may be made by its manufacturer, is not guaranteed or endorsed by the publisher.
